# Relationship of Interleukin 6 with Hepatic Steatosis and Liver Fibrosis in Rheumatoid Arthritis at a Rheumatology Care Center in Cartagena, Colombia

**DOI:** 10.3390/genes15121639

**Published:** 2024-12-21

**Authors:** Gloria Caterine Pérez-Mingan, Rita Magola Sierra-Merlano, Ismael Yepes, María Judith Palomino Vergara, Miguel Ortiz, Breiner Peña, Eder Cano-Pérez, Doris Gómez-Camargo

**Affiliations:** 1 Departamento de Medicina Interna, Facultad de Medicina, Universidad de Cartagena, Cartagena 130001, Colombia; gperezm1@unicartagena.edu.co (G.C.P.-M.); rmagola@unicartagena.edu.co (R.M.S.-M.); ismaelyb@unicartagena.edu.co (I.Y.); 2 Programa de Medicina, Facultad de Medicina, Universidad de Cartagena, Cartagena 130001, Colombia; mpalominov@unicartagena.edu.co; 3 Programa de Medicina, Facultad de Ciencias de la Salud, Universidad del Sinú, Cartagena 130001, Colombia; miguelortiz@unisinu.edu.co; 4 Programa de Medicina, Facultad de Ciencias de la Salud, Universidad del Magdalena, Santa Marta 470001, Colombia; breinerpenadp@unimagdalena.edu.co; 5 Grupo de Investigación UNIMOL, Facultad de Medicina, Universidad de Cartagena, Cartagena 130001, Colombia; ecanop@unicartagena.edu.co; 6 Doctorado en Medicina Tropical, Facultad de Medicina, Universidad de Cartagena, Cartagena 130001, Colombia

**Keywords:** rheumatoid arthritis, cytokines, interleukin 6, liver fibrosis

## Abstract

Background/Objectives: This study aimed to investigate the association of IL-6 with steatotic liver disease (SLD) and liver fibrosis (LF) in rheumatoid arthritis (RA) patients at a rheumatology center in Cartagena de Indias, Colombia. Methods: This was a cross-sectional study that included RA and non-RA cases. The level of cellular expression of interleukin 6 (IL-6) was evaluated by flow cytometry in peripheral blood leukocytes, and the presence of SLD and LF was detected by elastosonography. The main outcome was to establish the association between the levels of cellular expression of IL-6 and the development of SLD and LF. Results: This study included 47 cases of RA and 34 cases on-RA, with a mean age of 54 and 55 years, respectively. The frequency of SLD was 55.3% in RA and 38.2% in non-RA. The frequency of LF was 12.8% in RA and 14.7% in non-RA, with no statistical difference. The levels of cellular expression of IL-6 were significantly higher in RA compared to non-RA. Cellular expression of IL-6 was associated with the presence of SLD (54% vs. 30.3%; *p* = 0.002). This association was not maintained in RA cases (49.5% vs. 47.6%; *p* = 0.571). No association was found between cellular expression of IL-6 and LF in the total population (43.8% vs. 42.7%; *p* = 0.813) nor in RA cases (59.41% vs. 48.3%; *p* = 0.526). Conclusions: IL-6 levels were related to SLD in the evaluated sample, and RA was not a risk factor for SLD or LF. The prognostic role of IL-6 for SLD in patients with RA requires further studies.

## 1. Introduction

Rheumatoid arthritis (RA) represents a complex and impactful entity, characterized by its chronic, systemic, and autoimmune inflammatory nature that affects the joints. Its pathogenesis involves a complex interaction between genetic, proteomic, and environmental factors that trigger both innate and adaptive immune responses. This disorder primarily manifests as symmetric erosive polyarthritis and chronic synovitis [1,2].

In the context of musculoskeletal diseases (ME), RA occupies a prominent place, being the second most significant cause of years of life lost and years of productivity lost, after mental illnesses (DALYs). Within this spectrum of diseases, the burden of RA, along with others such as osteoarthritis (OA), substantially contributes to morbidity and loss of quality of life for affected individuals [3,4].

The prevalence of RA from 1986 to 2014 was 0.46% (95% CI 0.37–0.57%) and doubled from 2017–2021 [5]. In Colombia, the prevalence of RA in the study by the Colombian Association of Rheumatology (Asoreuma) is 1.49% (95% CI 1.12–1.98%), with 80.7% being women and a female-to-male ratio of 4.2:1. The prevalence of RA increases between the ages of 40 and 60 years [6]. In Colombia, the burden of ME is similar to other countries in the region, with RA being the third most common after low back pain and OA [7].

RA not only affects the joints but can also manifest with extra-articular complications in up to 50% of cases, which is associated with increased morbidity and mortality in patients. The treatment of RA involves the use of disease-modifying antirheumatic drugs (DMARDs), which can have adverse effects and sometimes mimic the extra-articular manifestations of the disease [8].

One of the least understood systemic complications of RA is hepatic involvement, which can vary widely in its frequency and presentation, occurring in 6% to 74% of cases. The histopathology of the liver in RA is heterogeneous and non-specific, including Kupffer cell hyperplasia, increased centrilobular deposits of lipofuscin, inflammatory cell infiltration of portal tracts, or foci of parenchymal or hepatic necrosis [9].

On the other hand, steatotic liver disease (SLD) refers to a broad spectrum of liver damage attributed to hepatocyte injury, inflammatory processes, and fibrosis. Its presentation ranges from mild to severe forms, such as non-alcoholic steatohepatitis, advanced fibrosis, cirrhosis, and liver failure. SLD is the manifestation of a multisystem disorder with heterogeneous causes, presentation, course, and outcomes and is the leading cause of chronic liver disease worldwide, affecting nearly 38% of the adult population, with its pathogenesis rooted in systemic metabolic dysfunction [10,11].

SLD represents the greatest burden of disease related to chronic liver disease, including cirrhosis and liver cancer [12]. The United Network for Organ Sharing (UNOS) in the United States reported that SLD is the second most common indication for liver transplantation [13,14]. In Cartagena de Indias (Colombia), where our study was conducted, Vélez and colleagues reported SLD in 30% of cirrhosis cases [15].

The frequency of SLD in RA is not well studied, having been reported in 31% of cases and 1.4% in advanced stages [16,17]. Zou Y-W and colleagues in a 2022 retrospective study with 513 RA cases showed 21.4% RA-SLD. In RA-SLD, 32.4% of cases had a high 10-year cardiovascular risk, 10.9% had cardiovascular events, and in SLD-fibrosis, worse outcomes were observed [18].

In RA-SLD, the pathogenic mechanisms of RA inflammation, including cytokines, are important, with IL-6 and TNF being the most studied [1,19]. Several studies have reported that IL-6 family cytokines participate in the liver’s homeostatic mechanisms, such as pathogen eradication, regeneration, inflammation, and initiation and progression of tumors [20,21,22,23].

SLD is common in other autoimmune diseases such as psoriasis (Ps), although there are few studies on its prevalence in psoriatic arthritis (PsA). A recent study shows that although SLD is more common in PsA, the prevalence of liver fibrosis and metabolic syndrome is similar in both PsA and PsO, highlighting that insulin resistance is a key factor in the development of SLD and FH in these patients [24].

Studies also show that chronic hepatic exposure to IL-6 promotes gluconeogenesis [21] and insulin resistance [22]. IL-6 could be associated with hepatic insulin resistance, altered lipid metabolism, and SLD [20]. In this context, this research aims to determine the association of IL-6 cellular expression with SLD and LF in RA in a population from Cartagena, Colombia.

## 2. Materials and Methods

### 2.1. Study Design and Population

An observational, cross-sectional, prospective study was conducted, with the sample obtained from RA cases treated at an outpatient rheumatology center in Cartagena de Indias, Colombia, between June 2023 and April 2024.

### 2.2. Selection Criteria and Data Collection

The selection criteria included individuals aged 18 to 65 years diagnosed with RA according to EULAR criteria [25], with a body mass index (BMI) between 18.5 and 29.9 kg/m^2^, who had signed the informed consent form. Exclusion criteria included those using IL-6-modifying pharmacological interventions, other hepatotoxic drugs, or substances, those consuming alcohol in excess (more than 10 g/day for women and more than 20 g/day for men), those diagnosed with other rheumatic diseases, prior diagnosis of one or more liver diseases (such as viral hepatitis, HIV infection, autoimmune liver diseases, or known genetic disorders), diabetes, dyslipidemia, or any other condition that, in the investigators’ opinion, could interfere with the study requirements. Selected control cases without rheumatoid arthritis also had to meet the inclusion (aged 18 to 65 years with a BMI between 18.5 and 29.9 kg/m^2^, who had signed the informed consent form) and exclusion criteria except for the diagnosis.

For all cases, data for analysis were collected from the patients’ medical records. Data related to demographic variables such as sex and age, clinical variables, comorbidities, and laboratory results pertaining to the study variables were obtained.

### 2.3. Diagnosis of SLD and FH by Elastography

Elastography was performed by a hepatologist experienced in this procedure at a diagnostic center specifically for this purpose. A standardized transient elastography device was used. For the detection of SLD, the controlled attenuation parameter (CAP) was identified, classifying it into three stages based on the degree of fat infiltration: S1: 238 to 260 dB/m, S2: 260 to 290 dB/m, and S3: 290 to 400 dB/m.

FH was determined by identifying elasticity values; the degrees of fibrosis were divided into four categories: F0–F1: < 7.6 kPa, F2: 7.7–9.4 kPa, F3: 9.5–14 kPa, and F4: greater than 14 kPa. Significant FH was defined as a stage greater than or equal to F2.

### 2.4. Quantification of IL-6

After elastography, peripheral blood samples were collected in EDTA tubes (2 to 3 mL per patient) and transported to the Molecular Research Unit (UNIMOL) laboratory at the University of Cartagena for processing. Invitrogen eBioscience reagents (Anti-Hu IL-6; Ref: 46-7069-42) were used to detect IL-6 expression levels in leukocytes following the manufacturer’s recommendations and using an Attune NxT flow cytometer. Briefly, 100 µL of whole blood was taken in a 1.5 mL microcentrifuge tube and incubated with 1.5 mL of freshly prepared red blood cell lysis buffer for 15 min at room temperature. After 15 min, the cells were washed twice with flow cytometry staining buffer, with each wash followed by centrifugation at 500× *g* for 5 min. After the last wash, the cell sediment was fully dissociated by inversion. The cells were permeabilized and incubated with Anti-Hu IL-6 for 15 min at room temperature before being fixed. Subsequently, they were washed, resuspended in staining buffer, and analyzed in the flow cytometer using the BL3 channel excited at a wavelength of 488 nm (blue laser). IL-6 expression is reported as the percentage of IL-6-expressing cells among the total evaluated cells (10,000 cells per case).

### 2.5. Statistical Analysis

The data were analyzed using the IBM^®^ SPSS^®^ statistical software, version 25. Quantitative and categorical variables were described using the mean (standard deviation) and percentages, respectively. The chi-square test and Student’s *t*-test were used for hypothesis testing and group comparisons, as appropriate. A *p*-value of 0.05 or less was considered statistically significant.

### 2.6. Ethical Considerations

This study was conducted in accordance with the ethical standards outlined in Resolution 008430 of 1993 issued by the Ministry of Health of Colombia. This project falls under the category of research with greater than minimal risk, considering it involved radiological studies with low-frequency microwaves, although to date, no complications from the procedure have been reported. It posed no real or potential risk.

Authorization from relevant authorities was obtained for the study. All precautions were taken to ensure the confidentiality of personal information; all participants provided informed consent for access to their medical records, for the collection of a peripheral blood sample, and for the performance of elastography. Data anonymization was carried out using data reduction techniques, eliminating particularly sensitive information that could serve as direct identifiers and was not relevant for analysis. A numeric code was assigned to identify each record as unique. None of the participating investigators declared any conflict of interest.

## 3. Results

**Figure 1:**
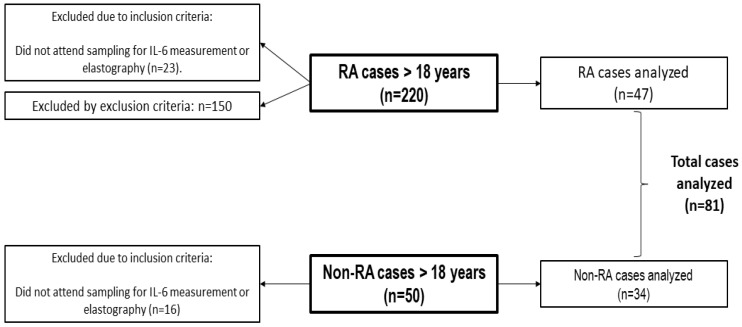
Flowchart of case selection for the study.

The study identified 220 RA cases and 50 non-RA cases, all with similar sociodemographic characteristics. For the final analysis, 47 RA cases and 34 non-RA cases were included, resulting in a total of 81 cases analyzed. Additional details of the participant selection and exclusion process are illustrated in Figure 1.

### 3.1. Comparative Analysis Between RA and Non-RA Cases

In RA cases, the mean age was 54 years (SD 9.1) vs. 55 years (SD 7.6) for non-RA cases, with a predominance of women in both groups (93.6% vs. 100%, respectively). Additionally, 100% (*n* = 34) of non-RA cases and 85.1% (*n* = 40) of RA cases came from Cartagena (Bolívar), with the remaining 14.9% coming from other municipalities in Bolívar. Osteoporosis was more common in RA cases compared to non-RA cases (19.1% vs. 0%; *p* = 0.007) and cellular expression levels of IL-6 were higher in RA cases vs. non-RA cases (48.7% vs. 34.7%; *p* = 0.000) (Table 1).

SLD was identified in 55.3% (*n* = 26) of RA cases and in 38.23% (*n* = 13) of non-RA cases; the most frequent stage for both groups was S3 (25.5% vs. 17.6%; *p* = 0.4). FH was found in 12.8% (*n* = 6) of RA cases and in 14.7% (*n* = 5) of non-RA cases; the most frequent stage in RA-FH was F1 (6.4%), and in non-RA-FH they were F2 (5.9%) and F3 (5.9%), with no significant differences (Table 2).

### 3.2. Factors Associated with SLD and FH in RA

In RA-SLD cases, the mean age was 54.1 years (SD 8.1) vs. 53.7 years (SD 10.4) in RA without SLD. Of the variables studied, the only one associated with SLD in RA was BMI (25.1 kg/m^2^ vs. 23 kg/m^2^, *p* = 0.011) (Table 3).

In RA-FH cases, the mean age was 60 years (SD 3.1) and in RA without FH it was 57 years (SD 4.4). Comorbidities and the use of DMARDs did not show statistical differences between the groups (Table 3).

### 3.3. Factors Associated with SLD and FH in All Cases

In all cases with a diagnosis of SLD, the mean age was 56 years (SD 8.1) vs. 53.8 years (SD 8.8) in the group without SLD (*p* = 0.306). No association was found between RA and SLD (66.7% vs. 50%; *p* = 0.129). On the contrary, BMI was significantly associated with SLD (BMI 25.2 kg/m^2^ vs. 23.4 kg/m^2^; *p* = 0.001) (Table 4).

In subjects with FH, no association was found between RA and FH (37.5% vs. 60.3%; *p* = 0.215); however, age was closely associated with FH in our population (60.8 years vs. 54 years; *p* = 0.032) (Table 4).

### 3.4. Association Between Cellular Expression of IL-6 and SLD-FH in All Cases

Cellular expression of IL-6 was associated with the presence of SLD (54% vs. 30.3%; *p* = 0.002). This association was not maintained in RA cases (49.5% vs. 47.6%; *p* = 0.571).

On the other hand, no association was found between cellular expression of IL-6 and FH in the total population (43.8% vs. 42.7%; *p* = 0.813) nor in RA cases (59.41% vs. 48.3%; *p* = 0.526) (Table 5 and Table 6).

## 4. Discussion

RA is a chronic rheumatic disease that constitutes a significant global disease burden measured by DALYs and is the third most burdensome disease in Colombia, following lumbago and OA [3,4,6,7]. In this study conducted in a predominantly female outpatient population from the Caribbean region of Colombia, SLD was identified in 55% of RA cases.

In 2020, Zamani M et al. reported a frequency of 35.3% for SLD in RA [26]. In 2022, Zou Y-W et al. observed a frequency of 21.4% for SLD in RA [18]. To date, we have not found studies on SLD in RA populations in Colombia. Differences in the frequencies observed between our results and other studies may be attributable to genetic, socioeconomic, and environmental factors.

Wong et al. [27] reported that SLD affects approximately 38% of adults worldwide. In Colombia, this disease affects around 26.6% [27,28]; findings consistent with our study, where we identified a frequency of 38% for SLD in non-RA cases.

Our results show significantly higher levels of cellular expression of IL-6 in RA, highlighting the role of this cytokine in the immunopathogenesis of inflammatory diseases, where its relationship with disease development and progression has been observed. These findings are consistent with those reported by Kavanaugh et al., who reported elevated levels of IL-6 not only in RA but also in psoriatic arthritis, ankylosing spondylitis, and other inflammatory diseases [29].

This study highlights the association between cellular expression levels of IL-6 and SLD, with our findings aligning with previous research that has suggested a relationship between IL-6 and hepatic inflammation processes and SLD development [19,30,31,32,33]. In Italy, Anna Alisi et al. reported significantly higher levels of IL-6 in obese populations with SLD [30]. Aysim Gunes et al. observed a significant increase in serum IL-6 levels in individuals with SLD, diabetes, and obesity [31]. In Spain, García-Galiano and colleagues documented elevated IL-6 levels in subjects with SLD, linking IL-6 as an independent prognostic factor for SLD [32]. In China, Duan Y. et al. noted a significant association between IL-6 levels and SLD [34].

When analyzing the RA population, cellular expression levels of IL-6 were not related to SLD. This finding is of particular interest and may be explained by the fact that, in addition to high cellular expression of IL-6, an individual genetic predisposition may also be required. Park et al. reported an association between the single nucleotide polymorphism (SNP) rs738409 C>G, which produces the genetic variant I148M. This variant was associated with increased SLD development and progression by elevating IL6/STAT3 signaling, suggesting a direct relationship between genetic factors and elevated levels of inflammatory cytokines [35].

Moreover, the finding of SLD and FH in subjects without risk factors supports the hypothesis of an underlying genetic predisposition and/or epigenetic mechanisms involved in the development of these conditions.

Finally, our study shows a significant association between body mass index (BMI) and the development of SLD. These findings are consistent with previous publications showing a strong and direct relationship between BMI and SLD [36,37,38].

Strengths of this study include its prospective design, being the first to explore biological markers in individuals with RA and SLD, and being the first Colombian study to establish the association between SLD and cellular expression of IL-6 in RA subjects. With this, it is possible to propose that there is an association between cellular expression of IL-6 and the development of SLD.

The findings in this study are important as they increase interest in research on measuring IL-6 in SLD and understanding the high economic and health burden and underlying cardiovascular disease risk associated with its diagnosis. Furthermore, the absence of commonly associated risk factors with SLD in this population highlights the need for further studies to identify the potential of cellular expression levels of IL-6 as a biomarker for SLD development and expands the need to explore genetic and/or epigenetic mechanisms involved in its development.

This research has certain limitations that should be considered when interpreting its findings. Firstly, the study was conducted with a small sample size, which may restrict the generalizability of the results to other populations. In addition, multivariate analysis was not included, which limits the ability to assess the possible causes of IL-6 elevation in patients with and without rheumatoid arthritis (RA). Also, the research was conducted in a single rheumatology referral center, which provides medical care to a population characterized by a high prevalence of poverty and belonging to a community with a similar culture.

## 5. Conclusions

IL-6 levels were associated with SLD in the evaluated sample; RA was not a risk factor for SLD or FH. The prognostic role of IL-6 for SLD in RA patients requires further studies.

## Figures and Tables

**Table 1:** Characteristics of cases with rheumatoid arthritis (RA) and without Rheumatoid Arthritis (Non-RA). Continuous variables are expressed as means (SD) and categorical variables as % (n).

Variable	RA (N = 47)	Non-RA (*n* = 34)
Age	54 (9.1)	55.5 (7.6)
Women	93.6 (44)	100 (34)
BMI (kg/m^2^).	24.1 (2.7)	24.5 (1.5)
Place of Origin		
Cartagena	85.1 (40)	100 (34)
Bayunca	4.3 (2)	0
Santa Rosa	2.1 (1)	0
Villa de Loro	2.1 (1)	0
Villanueva	2.1 (1)	0
Arenal	2.1 (1)	0
Turbaco	2.1 (1)	0
Arterial Hypertension	21.3 (10)	29.4 (10)
Heart Failure	2.1 (1)	0 (0)
Coronary Heart Disease	0 (0)	0 (0)
Osteoporosis	19.1 (9)	0 (0)
Depression	2 (4.3)	10.8 (4)
Smoking	8.5 (4)	0 (0)
IL-6	48.7 (11.3)	34.7 (10.2)

**Table 2:** Association between RA and steatotic liver disease/Hepatic Fibrosis.

Variable	RA (N = 47)	Non-RA (N = 34)	*p*-Value
Steatotic liver disease	55.3 (26)	38.23 (13)	0.129
S1	8.5 (4)	14.7 (5)	0.381
S2	21.3 (10)	5.9 (2)	0.054
S3	25.5 (12)	17.6 (6)	0.400
Hepatic fibrosis	12.8 (6)	14.7 (5)	0.8
F1	6.4 (3)	0 (0)	0.133
F2	4.3 (2)	5.9 (2)	0.739
F3	0 (0)	2.9 (1)	0.237
F4	2.1 (1)	5.9 (2)	0.377

**Table 3:** Factors Associated with Steatotic Liver Disease (SLD) and Hepatic Fibrosis (FH) in Cases with Rheumatoid Arthritis (RA).

Variable	RA-SLD	RA Without SLD	*p*-Value	RA-FH (*n* = 6)	RA Without FH (*n* = 41)	*p*-Value
Age	54.1 (8.1)	53.7 (10.4)	0.87	60 (3.1)	57 (4.4)	0.306
Women	92.3 (24)	95.2 (20)	0.683	83.3 (5)	95.1 (39)	0.270
BMI (kg/m^2^)	25.1 (2.6)	23 (2.6)	0.011	22.4 (2.4)	24.7 (2.3)	0.104
Comorbidities						
Arterial Hypertension	19.2 (5)	23.8 (5)	0.703	16.7(1)	22 (9)	0.768
Heart Failure	0 (0)	4.8 (1)	0.261	0	0	0
Osteoporosis	26.9 (7)	9.5 (2)	0.132	0	0	0
Depression	3.8 (1)	4.8 (1)	0.877	16.7 (1)	2.4 (1)	0.107
Smoking	0 (0)	19 (4)	0.020	16.7 (1)	7.3 (3)	0.443
DMARDs						
Metotrexate	80.8 (21)	76.2 (16)	0.703	66.7(4)	80.5 (33)	0.440
Leflunomida	26.9 (7)	42.9 (9)	0.252	16.7 (1)	36.6 (15)	0.336
Golimumab	0 (0)	9.5 (2)	0.108	0 (0)	4.9 (2)	0.580
Etanercept	7.6 (2)	0 (0)	0.108	16.6 (1)	2.4 (1)	0.102
Abatacept	0 (0)	4.8 (1)	0.261	0 (0)	2.4 (1)	0.699
Certolizumab	3.8 (1)	0 (0)	0.364	0 (0)	2.4 (1)	0.699
Corticoides	42.3 (11)	38.1 (8)	0.770	66.7 (4)	36.6 (15)	0.161
Years of Diagnosis	10.2 (4.9)	10 (7.3)	0.934	5 (4.7)	5 (4.3)	0.954
FIB4 Score	0.86 (0.54–1.1)	0.72 (0.4–1.09)	0.251	0.58 (0.32–0.92)	0.84 (0.49–1.2)	0.147
FR	68 (17)	71.4 (15)	0.801	66.7 (4)	70 (28)	0.869
Anti-CCP	42.9 (3)	16.7 (1)	0.308	16.6 (1)	7.3 (3)	0.377
AST	26.3 (19.3)	21.7 (8)	0.310	23.5 (8.5)	20.2 (5.5)	0.251
ALT	21.4 (8.7)	24.6 (15.4)	0.376	22 (20)	20 (7.5)	0.678
GGT	26.7 (10)	30 (17)	0.470	25 (8)	22 (8)	0.302
Platelets	340.500 (87.9)	325.571(102.667)	0.594	356.500 (77.236)	330.512 (96.624)	0.533
HbA1C	5.4 (0.43)	5.3 (0.44)	0.624	5.3 (0.40)	5.4 (0.48)	0.834
PCR	10.7 (19.4)	14.5 (18)	0.510	4.7 (9.2)	6 (9.8)	0.544
VSG	33.2 (16)	35.8 (21.6)	0.647	25.9 (11.8)	35.1 (19)	0.495
DAS-28	3.7 (1.1)	3.7 (1.6)	0.851	4.1 (1.65)	3.67 (1.3)	0.428
HAQ	0.24 (0.27)	0.1 (0.2)	0.362	0.24 (0.27)	0.1 (0.2)	0.362

**Table 4:** Factors associated with SLD and FH in all cases.

Variable	SLD (*n* = 39)	Non-SLD (*n* = 42)	*p*-Value	Significant FH (*n* = 8)	Without Significant FH (*n* = 73)	*p*-Value
Age	56 (8.1)	53.4 (8.8)	0.306	60.8 (4.7)	54 (8.6)	0.032
Women	94.9 (37)	97.1 (41)	0.513	100 (8)	95.9 (4.1)	0.559
BMI (kg/m^2^)	25.2 (2.2)	23.4 (2.1)	0.001	24.2(2.1)	24.3 (2.3)	0.871
Comorbidities						
RA	66.7 (26)	50 (21)	0.129	37.5 (3)	60.3 (44)	0.215
Arterial Hypertension	25.6 (10)	23.8 (10)	0.849	25 (2)	24.7 (18)	0.983
Heart Failure	0 (0)	2.4 (1)	0.332	0 (0)	1.3 (1)	0.739
Smoking	0 (0)	9.5 (4)	0.048	12.5 (1)	4.1 (3)	0.298
Depression	5.1 (2)	4.8 (2)	0.939	12.5 (1)	4.1 (3)	0.298
AST	26.3 (19.3)	21.7 (8.1)	0.310	27.3 (6.6)	24.1 (15)	0.731
ALT	21.4 (8.7)	24.6 (15.4)	0.376	24 (20)	22.7 (11.7)	0.869
GGT	26.7 (10)	30 (17.1)	0.731	43.5 (26.1)	27.1 (12.1)	0.087
Platelets	340.500 (87.957)	325.571 (101.000)	0.594	336.700 (62.268)	331.113 (957.178)	0.454
HbA1C	5.4 (0.43)	5.3 (0.44)	0.834	5.6 (0.3)	5.3 (0.42)	0.426
PCR	10.7 (19.4)	15.4 (18.6)	0.554	6 (11.4)	12.08 (19.4)	0.591
VSG	32.2 (16)	35.8 (21.4)	0.647	37.6 (11.8)	34.1 (19)	0.757
FIB 4 Score	0.86 (0.31)	0.72 (0.37)	0.251			

**Table 5:** Relationship between cellular expression of IL-6 and the presence of steatotic liver disease and liver fibrosis in RA.

IL-6	RA-SLD	RA-Non-SLD	*p*-Value
Percentage of Cellular Expression of IL-6 (SD)	49.5 (11.6)	47.6 (11.1)	0.571
IL-6	RA-FH	RA–Non-FH	*p*-value
Percentage of Cellular Expression of IL-6 (SD)	51.49 (2.69)	48.3 (12)	0.526

SD: Standard deviation.

**Table 6:** Cellular expression of IL-6 in relation to steatotic liver disease and liver fibrosis in all cases.

IL-6	SLD	Non-SLD	*p*-Value
Percentage of Cellular Expression of IL-6 (SD)	54 (2.6)	30.3 (12)	0.002
IL-6	FH	Non-FH	*p*-value
Percentage of Cellular Expression of IL-6 (SD)	43.8 (8.4)	42.7 (13.28)	0.813

SD: Standard deviation.

## Data Availability

All data used to support our findings are included in the manuscript. The corresponding author may provide any additional requested information.
